# Comprehensive Ethnopharmacological Analysis of Medicinal Plants in the UAE: *Lawsonia inermis*, *Nigella sativa*, *Ziziphus spina-christi*, *Allium cepa*, *Allium sativum*, *Cymbopogon schoenanthus*, *Matricaria aurea*, *Phoenix dactylifera*, *Portulaca oleracea*, *Reichardia tingitana*, *Salvadora persica*, *Solanum lycopersicum*, *Trigonella foenum-graecum*, *Withania somnifera*, and *Ziziphus lotus*

**DOI:** 10.3390/nu17030411

**Published:** 2025-01-23

**Authors:** Razan S. Almasri, Alaa S. Bedir, Seham M. Al Raish

**Affiliations:** 1 Department of Nutrition, College of Medicine and Health Science, United Arab Emirates University, Al Ain 15551, United Arab Emirates; 201950110@uaeu.ac.ae (R.S.A.); 201950078@uaeu.ac.ae (A.S.B.); 2 Department of Biology, College of Science, United Arab Emirates University, Al Ain 15551, United Arab Emirates

**Keywords:** bioactive compounds, dietary supplements, global nutrition, health promotion, medicinal plants, nutritional phytochemistry

## Abstract

The United Arab Emirates (UAE) is home to diverse indigenous medicinal plants traditionally used for centuries. This study systematically evaluates the pharmacological and nutritional potential of key medicinal plants, including *Lawsonia inermis*, *Nigella sativa*, *Ziziphus spina-christi*, *Allium cepa*, *Allium sativum*, *Cymbopogon schoenanthus*, *Matricaria aurea*, *Phoenix dactylifera*, *Portulaca oleracea*, *Reichardia tingitana*, *Salvadora persica*, *Solanum lycopersicum*, *Trigonella foenum-graecum*, *Withania somnifera*, *and Ziziphus lotus.* Comprehensive literature searches were conducted using PubMed, Scopus, and Web of Science to identify studies relevant to their nutritional and pharmacological uses. The findings highlight the therapeutic roles of these plants in managing global health challenges such as gastrointestinal diseases, and antimicrobial resistance through bioactive compounds like flavonoids, polyphenols, and antioxidants. Additionally, their contributions to nutrition, including essential vitamins and minerals, are emphasized for disease prevention and health promotion. While this research focuses on the UAE, the implications are globally relevant, as many of these plants are also found in traditional medicine across Asia, Africa, and Europe. Integrating these findings into global nutritional and healthcare systems offers potential solutions for pressing public health concerns, reduces reliance on synthetic pharmaceuticals, and promotes sustainable healthcare practices. This work is a valuable reference for researchers, healthcare professionals, and policymakers, bridging traditional knowledge and modern scientific applications globally.

## 1. Introduction

Medicinal plants (MPs) have historically been integral to both traditional and contemporary medicine globally, providing a variety of therapeutic advantages [1,2,3,4]. Additionally, ginger has long been utilized in traditional Chinese medicine to alleviate nausea and inflammation due to its anti-inflammatory and antioxidant properties [5]. Another example is the use of turmeric in Indian Ayurvedic medicine, where its active compound, curcumin, has been shown to have anti-inflammatory and antioxidant effects, making it a popular remedy for various ailments [6,7]. Furthermore, ginkgo tree leaves have been used in traditional Chinese medicine to improve memory and cognitive function, attributed to their ability to increase blood flow to the brain [8]. However, not all medicinal plants are safe or effective for all individuals. For instance, the herb comfrey was once commonly used for its purported healing properties, but studies have shown that it can cause liver damage and should be avoided [9,10]. Furthermore, some medicinal plants can interact negatively with certain medications, highlighting the importance of consulting a healthcare provider before incorporating them into one’s treatment regimen [11]. In recent years, many individuals have reported improvements in their symptoms and overall health following the use of medicinal plants. Research has also suggested that certain medicinal plants have anti-inflammatory and antioxidant properties, and there has been a significant increase in interest in plant-based therapies as individuals and researchers pursue alternative and complementary approaches for chronic health issues [12,13], primarily metabolic disorders such as diabetes, cardiovascular diseases, and infections resistant to antimicrobials [14,15]. Plant-based therapies have shown promising results in managing chronic health issues and the antimicrobial properties of plants that can help combat these. As more studies are conducted to explore the potential benefits of plant-based therapies, individuals need to work closely with healthcare providers to ensure the safe and effective integration of these treatments into their healthcare regimens. By working with healthcare providers, individuals can receive personalized guidance on the best plant-based therapies for their conditions and medical history. Patients must communicate openly with their healthcare team about any plant-based treatments they are considering, as certain herbs and supplements may interact with medications or exacerbate existing health issues. With the proper guidance and monitoring, incorporating plant-based therapies into one’s healthcare regimen can be a valuable addition to conventional treatments for chronic health conditions. The United Arab Emirates (UAE) possesses distinctive desert vegetation and a profound history of traditional medicine, offering a significant collection of culturally essential and pharmacologically valuable medicinal plants [16,17,18]. The incidence of diabetes and cardiovascular diseases in the UAE is increasing [19], mirroring global patterns linked to lifestyle modifications and urbanization [20,21]. Given the increasing prevalence of chronic health conditions such as diabetes and cardiovascular diseases in the UAE, incorporating plant-based therapies into healthcare regimens could provide valuable support alongside conventional treatments. The UAE’s unique desert vegetation and traditional medicine practices offer a rich source of culturally significant and pharmacologically potent medicinal plants. By combining modern medical guidance with the use of these natural remedies, individuals in the UAE can take proactive steps towards managing their health in a holistic manner that aligns with global trends towards lifestyle modifications and urbanization. The concurrent challenge of antimicrobial resistance prompts an urgent quest for innovative solutions, rendering plant-derived antimicrobials a compelling research focus [22]. The native and adapted florae of the UAE, recognized for their resilience to extreme climatic conditions, possess bioactive compounds that may effectively address and alleviate these health issues [23,24]. By tapping into the abundant natural remedies in the UAE’s florae, individuals worldwide can embrace a holistic approach to health management that mirrors global trends. The investigation into plant-derived antimicrobials in the region is a fascinating example of innovative solutions that could combat antimicrobial resistance on a worldwide scale. With their resilience to extreme conditions, the florae of the UAE present promising opportunities for addressing various health issues and promoting overall well-being.

This review consolidates the available scientific literature on MPs in the UAE, focusing on those with documented gastrointestinal disease and antibacterial properties. It aims to underscore the significance of these plants’ pharmacological profiles and potential therapeutic applications in tackling the UAE’s urgent health challenges. The review also addresses the difficulties related to the standardization, preservation, and sustainable utilization of MPs, which are essential for guaranteeing that these resources can support enduring health solutions [25,26]. This study enhances the global understanding of ethnopharmacology and promotes additional investigation into the UAE’s botanical assets. It could potentially result in the creation of effective plant-derived therapeutic agents that conform to sustainable healthcare principles.

## 2. Methodology

### 2.1. Selection Criteria

#### 2.1.1. Data Collection

Between June and December 2024, a comprehensive analysis of UAE florae was conducted using multiple references, including digital sources. Following the compilation of botanical names and synonyms, we performed an exhaustive literature review for each plant. This procedure utilized online resources, such as Google Scholar and scientific databases like PubMed, to acquire articles concerning plants that are native to or naturalized in the UAE and their medicinal uses. The search employed keywords that combined each plant’s botanical nomenclature or synonyms with its therapeutic uses for diabetes, antibacterial properties, and cardioprotective effects. In conducting this comprehensive review, meticulous attention was dedicated to the selection and analysis of relevant data to ensure the robustness and relevance of our findings. Recognizing the historical depth of medicinal plant research, our criteria included seminal studies dating beyond the last two decades, thus embracing foundational works that have shaped current understanding. To quantitatively assess the pharmacological efficacy of the selected plants, we calculated effect sizes using eta squared (η^2^) statistics and visually represent these findings through detailed forest plots. This methodological rigor enhances the reliability of our review and supports the integration of traditional uses of medicinal plants with evidence-based scientific research, providing a well-rounded perspective on their potential health benefits.

#### 2.1.2. Inclusion Criteria

The research focused on medicinal plants native to or commonly utilized in the UAE, We initially included 15 widely recognized medicinal plants in this study, ranging from global staples like onion and garlic to locally significant species such as the date palm and non-native agriculturally important plants like tomatoes, emphasizing their relevance in traditional and modern medicine. The scope was narrowed explicitly to studies examining these plants’ antidiabetic, cardiovascular, or antimicrobial properties. The selected literature comprises peer-reviewed articles, including experimental research (in vitro and in vivo studies), observational studies, ethnobotanical surveys, and reviews published in reputable journals. To ensure the inclusion of current data, the research incorporated studies published within the last 20 years. Furthermore, the chosen studies provide comprehensive pharmacological or phytochemical evidence detailing active compounds, mechanisms of action, and efficacy in supporting the medicinal potential of these plants. The literature search was conducted from July to December 2024 to ensure the most recent and relevant studies were included.

#### 2.1.3. Exclusion Criteria

We omitted medicinal plants that are neither indigenous to nor commonly utilized in the UAE, as well as those lacking particular significance to the UAE’s traditional medicine. Articles that exclusively addressed plants’ nutritional, agricultural, or ornamental attributes without any reference to medicinal or health-related applications were also excluded. Furthermore, non-peer-reviewed sources, such as anecdotal evidence, unverified assertions, and publications from journals lacking scientific rigor were excluded to maintain the integrity of the research. A language restriction was applied to include only studies published in English unless a reliable translation were available, providing consistent analysis and accurate interpretation of the data. Studies that did not specifically examine antidiabetic, cardiovascular, or antibacterial effects or lacked pertinent data on these health outcomes were also omitted from the analysis.

### 2.2. Effect Size Calculation

Effect size was estimated using IBM SPSS version 30 for Mac OS, and forest plots was generated using JASP version 0.19 2024.

## 3. Medicinal Plants in the UAE—Overview

### 3.1. Allium cepa

*Allium cepa* (*A. cepa*), commonly known as onion, is a widely cultivated and consumed annual or biennial plant belonging to the genus *Allium* of the Amaryllidaceae family. It is typically cultivated in fields from October to December [27]. This dietary fiber-rich plant is recognized as both a popular condiment and a nutritional powerhouse, containing high levels of folic acid and vitamin B6 and essential minerals such as magnesium, calcium, potassium, and phosphorus [15,28]. Beyond its culinary uses, *A. cepa* has been extensively studied for its therapeutic properties. Research highlights its antimicrobial activity alongside its anticancer, antidiabetic, antioxidant, antiplatelet, antihypertensive, and antidepressant effects [28,29]. Additionally, *A. cepa* demonstrates neuroprotective, anti-inflammatory, cardioprotective, and antiparasitic properties, with positive impacts on the digestive, circulatory, respiratory, cardiac, and immune systems [28,30].

### 3.2. Allium sativum

*Allium sativum* (*A. sativum*), commonly known as garlic, is a widely consumed aromatic plant with a rich history in traditional medicine and cuisine globally [31,32]. Belonging to the genus *Allium* of the Amaryllidaceae family, it is planted in UAE nurseries during November to take advantage of the optimal growing conditions, although the full cultivation cycle extends beyond this month. Rich in sulfur-containing compounds such as alliin, allicin, and ajoene, as well as flavonoids like quercetin, *A. sativum* is renowned for its therapeutic properties, including anticarcinogenic, antioxidant, antidiabetic, renoprotective, anti-atherosclerotic, antimicrobial, anti-obesity, and antihypertensive effects [32,33,34]. Its active components, particularly allicin, exhibit antibacterial, antiviral, antifungal, anti-inflammatory, and anticancer activities, contributing to its efficacy in immunomodulation and cytokine regulation [15,33].

### 3.3. Cymbopogon schoenanthus

*Cymbopogon schoenanthus* (*C. schoenanthus*), commonly known as lemongrass, is a perennial plant native to the UAE from the genus *Cymbopogon* of the family Poaceae. It grows on rocky grounds and roadsides [35]. Methanolic extracts of *C. schoenanthus* contain tannins, steroids, alkaloids, essential oils, terpenoids, and flavonoids, and demonstrate significant antibacterial activity against foodborne pathogens [36,37].

### 3.4. Lawsonia inermis

*Lawsonia inermis* (*L. inermis*), commonly known as henna, is a perennial plant native to the UAE, where it thrives in sandy soils and oasis areas. Traditionally cultivated, its leaves, leaf juice, and oil extracts are used for their antimicrobial properties [35,38]. *L. inermis* is widely recognized in Persian medicine for its therapeutic potential [39,40].

*L. inermis* is a medicinal plant rich in bioactive compounds, including carbohydrates, flavonoids, phenolics, proteins, saponins, terpenoids, alkaloids, xanthones, resins, quinones, coumarins, fats, and tannins. Key constituents such as 2-hydroxy-1,4-naphthoquinone (lawsone) and gallic acid are particularly noteworthy for their pharmacological activities [39]. The plant exhibits a wide range of biological properties, including antibacterial, antifungal, antiparasitic, anti-inflammatory, analgesic, wound-healing, and anticancer effects. Different parts of the plant, including roots, leaves, fruits, stems, and flowers, have been utilized in traditional medicine to treat various ailments [39].

### 3.5. Matricaria aurea

*Matricaria aurea* (*M. aurea*), also known as golden chamomile, is a widely used medicinal plant native to the UAE, commonly found in rocky areas, mountains, and fields. Flowering from February to April, its extracts exhibit analgesic, anti-inflammatory, antibacterial, and antioxidant properties [41,42].

### 3.6. Nigella sativa

*Nigella sativa* (*N. sativa*), or black cumin, is an annual plant cultivated on private farms in the UAE. Known for its antioxidant and anti-inflammatory properties, it has shown efficacy in managing diabetes, hypertension, and hypercholesterolemia, as well as exhibiting anticancer and immunomodulatory activities [24,43].

### 3.7. Phoenix dactylifera

*Phoenix dactylifera* (*P. dactylifera*), or date palm, is a perennial tree native to the UAE. Tolerant to salinity, it is cultivated throughout the country and provides a wide range of bioactive compounds, including flavonoids, phenolic acids, and carotenoids. The plant exhibits hepatoprotective, nephroprotective, antioxidant, and anticancer activities, and contributes to fertility health [44].

### 3.8. Portulaca oleracea

*Portulaca oleracea* (*P. oleracea*), commonly known as purslane, is an annual plant native to the UAE, often found in valleys and roadside areas. Known for its rich composition of omega-3 fatty acids, flavonoids, and phenolic compounds, it has demonstrated antibacterial, antioxidant, and anticancer activities [45,46].

### 3.9. Reichardia tingitana

*Reichardia tingitana* (*R. tingitana*), or poppy-leafed Reichardia, is native to the UAE and thrives in rocky and mountainous regions. Extracts of this annual plant are rich in phenolics, tannins, flavonoids, and essential oils, showcasing antimicrobial and cytotoxic properties [12,47]. *R. tingitana* has been extensively studied for its phytochemical composition and diverse biological activities. Its extracts exhibit significant antimicrobial properties [12]. Various bioactive compounds have been isolated from the aerial parts of *R. tingitana*, including phenolics, tannins, flavonoids, coumarins, phytosterols, lactones, and essential oils [12,47,48]. Quantitative analysis of the shoot extracts revealed high concentrations of phenolics (143.68 mg gallic acid equivalent/g dry extract), flavonoids (32.41 mg catechin equivalent/g dry extract), and tannins (96.57 mg gallic acid equivalent/g dry extract) [47].

### 3.10. Rhazya stricta

*Rhazya stricta* (*R. stricta*) is a perennial shrub from the genus *Rhazya* of the Apocynaceae family, native to the UAE and widely distributed across Arabian countries [35,49,50]. It thrives in sandy, gravelly soils, rocky areas, and wadi beds, with flowering occurring between February and June [35]. Traditionally, *R. stricta* has been utilized to treat various ailments, including diabetes mellitus, syphilis, parasitic infections, rheumatism, and the common cold [49,51]. The plant’s leaf extracts, rich in glycosides, alkaloids, tannins, and triterpenes, exhibit potential therapeutic effects for diabetes, cancer, and inflammation [50].

### 3.11. Salvadora persica

*Salvadora persica* (*S. persica*), commonly known as *miswak* or *siwak*, is a perennial plant from the genus *Salvadora* of the Salvadoraceae family [52]. It is widely cultivated in the UAE, particularly in sandy regions, western dunes, wadis, and coastal areas, where it also serves as a windbreak [35]. Known as the *arak* or toothbrush tree, *S. persica* has a long history of use in oral hygiene, with its roots, twigs, and stems employed as natural cleaning tools [35,53]. Studies have demonstrated its antiplaque, anticaries, anti-inflammatory, and antimycotic properties, largely attributed to benzyl isothiocyanate, a compound that inhibits lactic acid production and the growth of *Streptococcus mutans (S. mutans)* [54].

Complementing this, Kumar and Sharma (2021) provided a review of the traditional uses and pharmacological activities of miswak, identifying its efficacy in treating a range of conditions from dental health to systemic diseases like diabetes and arthritis due to its diverse phytochemical content, which includes alkaloids, flavonoids, and terpenoids [55]. Abdel-razakh et al. (2024) explored the antioxidant properties of *S. persica*, among other plants, in traditional African medicine, indicating its limited antioxidant activity, but the potential for other bioactive effects [56].

### 3.12. Solanum lycopersicum

*Solanum lycopersicum* (*S. lycopersicum*) (tomato) is an annual plant from the genus *Solanum* of the Solanaceae family, widely cultivated on farms and in home gardens throughout the UAE. Its flowering period extends from March to December [35]. As one of the most consumed crops globally, tomatoes are valued for their micronutrients and bioactive compounds, including carotenoids, polyphenols, tocopherols, terpenes, and sterols [57]. These compounds, which retain their health-promoting properties after industrial processing, are associated with reduced risks of chronic diseases such as cancer, cardiovascular diseases, and type 2 diabetes [58,59].

### 3.13. Trigonella foenum-graecum

*Trigonella foenum-graecum* (*T. foenum-graecum*), common name fenugreek, is an annual plant from the genus *Trigonella* of the Fabaceae family [35]. Cultivated in sandy, silty, and clay soils in the UAE, fenugreek seeds have long been valued for their medicinal properties across cultures [60,61]. Rich in bioactive compounds such as saponins, galactomannans, and trigonelline, fenugreek seeds are recognized for their antidiabetic, hypocholesterolemic, and antibacterial activities [62,63]. Germinated seeds in particular have shown efficacy in treating *E. coli* infections, highlighting the plant’s therapeutic potential [62].

Fenugreek is noted for its wide-ranging pharmacological effects, such as antidiabetic, anti-inflammatory, and hypocholesterolemic properties, as detailed by Bahmani et al. (2016) and Yadav and Baquer (2014), who describe its use in enhancing metabolic health and managing blood lipid levels. Collectively, these studies present *T. foenum-graecum* as a multifaceted herbal remedy with profound benefits across various aspects of human health, advocating for its inclusion in pharmacological and dietary strategies for comprehensive wellness and disease management [64,65].

### 3.14. Withania somnifera

*Withania somnifera* (*W. somnifera*), common name ashwagandha, is a perennial shrub from the genus *Withania* of the Solanaceae family, thriving in sandy soils and waste areas in the UAE [35,66]. Flowering during the summer, this medicinal plant has garnered attention for its adaptogenic, anti-inflammatory, and antidiabetic properties [66,67]. Phytochemical analyses reveal that its roots and leaves contain bioactive compounds such as withanolides, saponins, and flavonoids, which contribute to its effectiveness in treating neurological disorders, anxiety, and hyperlipidemia [68,69].

### 3.15. Ziziphus lotus

*Ziziphus lotus* (*Z. lotus*), commonly known as *sedra*, is a perennial plant from the genus *Ziziphus* of the Rhamnaceae family, thriving on stony slopes and alluvial plains in the UAE [35,70]. Flowering from March to April, *Z. lotus* is rich in secondary metabolites such as phenolics, sterols, and tocopherols, which contribute to its antioxidant, anti-inflammatory, antidiabetic, and antispasmodic activities [71,72]. Despite its therapeutic potential, the less polar fractions of *Z. lotus* remain underexplored, presenting opportunities for future research in natural product development [73].

## 4. Overview of Antibacterial Properties of Medicinal Plants in the UAE

### 4.1. Allium cepa

*A. cepa* extracts have demonstrated significant antimicrobial properties, particularly against pathogenic bacteria. Subcritical water extracts (SWEs) of onion peel showed superior antimicrobial effects compared to methanol extracts, effectively reducing cell growth in various strains of *S. aureus* (KCCM 40510, KCCM 32395, KCCM 11335) by 0.7–1.1 log CFU/mL. Although SWE exhibited slightly lower antimicrobial effectiveness than pure quercetin (used as a standard), this difference is attributed to the lower quercetin concentration in the extracts. The antimicrobial mechanism involves disrupting bacterial energy metabolism, cytoplasmic membrane functions, and nucleic acid biosynthesis. These findings highlight the potential of *A. cepa* extracts [74,75], particularly onion peel [76,77], as natural antimicrobial agents with applications in food preservation and antibacterial treatment development.

Furthermore, onion peel waste has demonstrated significant potential in the green synthesis of metal nanoparticles, such as gold and silver, with diverse applications. These nanoparticles show promise in the biomedical field for developing antibacterial drugs and wound dressings. The food industry can utilize these nanoparticles to create antimicrobial coatings and innovative packaging solutions. Their wide applicability underscores the versatility and sustainability of onion peel-derived nanoparticles in various sectors [78].

### 4.2. Allium sativum

*A. sativum* contains a diverse array of bioactive compounds, including oil-soluble organosulfur compounds such as allicin, ajoene, and allyl sulfides, as well as water-soluble compounds [79,80] like allicin, a key bioactive compound in garlic that is formed when it is enzymatically converted by alliinase upon crushing or chopping garlic. Allicin not only contributes to garlic’s characteristic odor but also exhibits potent antimicrobial, antifungal, antiviral, and antioxidant properties [81,82,83,84].

Garlic’s antimicrobial activity is attributed to its ability to disrupt bacterial cell membranes, inhibit enzyme activity, and interfere with microbial metabolism. Studies have demonstrated its efficacy against a wide range of Gram-positive and Gram-negative bacteria, including *E. coli*, *Salmonella enterica* (*S. enterica*), *Shigella*, *Listeria monocytogenes* (*L. monocytogenes*), *Pseudomonas aeruginosa* (*P. aeruginosa*), *S. aureus*, and *Clostridium difficile*. Ethanolic garlic extract (EGE) has also shown effectiveness against multidrug-resistant strains, including methicillin-resistant *S. aureus* (MRSA) and vancomycin-resistant *Enterococcus* [81,85].

Allicin’s antimicrobial properties extend to its synergistic effects with other bioactive compounds. For instance, ajoene, a derivative of allicin, enhances garlic’s antibacterial potential. Garlic extracts, including fresh garlic extract and garlic oil, have been found effective against both commensal and pathogenic bacteria, including *Helicobacter pylori* (*H. pylori*), with the ability to reduce bacterial colonization in the gastrointestinal tract.

In addition to its antimicrobial properties, *A. sativum* exhibits significant antifungal activity, particularly against *Candida albicans* (*C. albicans*) and other fungal pathogens [86,87,88]. Its bioactive components disrupt fungal cell walls and inhibit spore formation, making it a potential alternative for managing fungal infections [89,90,91]. Moreover, garlic has demonstrated antiviral activity against influenza viruses and herpes simplex viruses.

Garlic’s diverse therapeutic properties, particularly its ability to target drug-resistant pathogens, highlight its potential as a natural source for novel antimicrobial agents. Its broad-spectrum activity, combined with its low toxicity and affordability, underscores its value in both traditional and modern medicine [92,93,94,95,96,97]. Further research is warranted to explore the clinical applications of *A. sativum* in treating infections and other health conditions.

Research highlights garlic’s broad-spectrum antibacterial activity, effective against both Gram-negative and Gram-positive bacteria, such as *E. coli*, *Salmonella*, *Shigella*, *S. aureus*, *P. aeruginosa*, and *L. monocytogenes* [81]. EGE has shown efficacy against multidrug-resistant pathogens, including Mycobacterium tuberculosis and vancomycin-resistant *S. aureus* [81]. Additionally, garlic’s antibacterial mechanism is attributed to allicin, which disrupts essential metabolic processes by interacting with thiol-containing enzymes such as thioredoxin reductase and ribonucleic acid (RNA) polymerase. Garlic extracts also inhibit bacterial toxin production and prevent the growth of enterotoxigenic *E. coli* and other intestinal pathogens [33].

### 4.3. Cymbopogon schoenanthus

*C. schoenanthus* is rich in bioactive compounds such as saponins alongside essential minerals like iron, sodium, and potassium [37]. Traditionally used in some countries’ medicine as an anti-infective for urinary tract infections and a diuretic for kidney stone prevention [98,99], *C. schoenanthus* demonstrates effective antibacterial activity [37,100,101]. Its methanolic extracts exhibit concentration-dependent inhibition against pathogens like *B. cereus*, *S. aureus*, *E. coli.*, *Klebsiella pneumoniae* (*K. pneumoniae*), and *Escherichia coli* (*E. coli*) [36,37].

Additionally, the essential oil of *C. schoenanthus* has shown efficacy against methicillin-sensitive and MRSA and other bacterial pathogens, making it a promising natural source for antimicrobial applications [100,102,103].

### 4.4. Lawsonia inermis

Chloroform extracts of *L. inermis* leaves have shown antibacterial activity against nosocomial pathogens such as *S. aureus* and *K. pneumoniae*, with minimum inhibitory concentrations (MICs) of 100 mg/mL and 200 mg/mL, respectively. Similarly, methanolic extracts have demonstrated efficacy against *E. coli*, and *S. aureus* at similar MIC values [104]. Furthermore, the combination of *L. inermis* extracts with *Lactobacillus plantarum* has exhibited synergistic effects in wound healing by reducing the inflammatory markers interleukin 6 (IL-6) and tumor necrosis factor α (TNF-α), promoting faster recovery in infected wounds [105].

*L. inermis* has also been explored for its antifungal properties, particularly against *C. albicans* and dermatophytic fungi [86,106]. Its wound-healing properties are attributed to its ability to reduce oxidative stress and inflammation, making it a promising candidate for natural therapeutic applications [98,107,108]. The plant’s diverse bioactive compounds contribute to its potential in addressing resistant infections, especially as an alternative to synthetic antimicrobial agents.

These findings highlight the therapeutic potential of *L. inermis* as a natural source of antimicrobial and wound-healing agents. Additional research is necessary to investigate its clinical applications in contemporary medicine.

### 4.5. Matricaria aurea

*M. aurea* contains a complex profile of bioactive compounds, including α-bisabolol oxide A, chlorogenic acid, and ferulic acid [42,109]. Essential oils and methanolic fractions of *M. aurea* have demonstrated antibacterial activity against *Bacillus subtilis* (*B. subtilis*), *S. aureus*, and *P. aeruginosa* [110,111,112,113]. The plant’s extracts effectively reduce bacterial adhesion, making them suitable for treating periodontal diseases and biofilm-associated infections [42,114]. These findings highlight the potential of *M. aurea* as a natural therapeutic agent in oral health and infectious disease treatment [42,113,114]. Additionally, studies have shown that *M. aurea* extracts possess anti-inflammatory properties, further supporting their potential in managing oral health conditions. Further research is warranted to explore the full range of therapeutic benefits that this plant may offer in the field of medicine [114,115,116].

### 4.6. Phoenix dactylifera

*P. dactylifera* has a rich array of bioactive compounds, including proanthocyanidins [117]. The methanolic extract of date palm exhibits antibacterial activity against *E. coli*, *S. aureus*, and *B. cereus* [118,119,120]. Additionally, date extracts possess antioxidant [121,122], anti-inflammatory [44,123,124], and anticancer [125,126] properties, emphasizing their potential as a natural source for therapeutic applications.

### 4.7. Portulaca oleracea

*P. oleracea* is a medicinal plant with significant antioxidant and antimicrobial properties, attributed to its rich phytochemical profile. Bioactive compounds such as ascorbic acid, α-tocopherols, omega-3 fatty acids, apigenin, gallotannins, quercetin, and kaempferol contribute to its diverse pharmacological activities [127]. Purslane has demonstrated broad-spectrum antibacterial activity against Gram-positive and Gram-negative bacteria, including *P. aeruginosa*, *Neisseria gonorrhea*, *E. coli*, *Streptococcus faecalis*, *Bacillus* species, and *S. aureus* [127]. Additionally, a lectin isolated from the roots of Portulaca elatior exhibited bacteriostatic activity against *Enterococcus faecalis* (*E. faecalis*), *P. aeruginosa*, and *S. aureus* [128].

Research highlights the diverse therapeutic potential of Portulaca species, particularly *P. oleracea*. A study by He et al. (2023) demonstrated the clinical efficacy of *P. oleracea* in treating bacterial diarrhea and its anti-inflammatory effects, attributed to its ability to regulate gut microbiota and fecal metabolism [129]. Liu et al. (2023) reported that organic acid extracts of purslane exhibit specific *Staphylococcus*-killing properties [128]. Soliman et al. (2017) found that purslane extracts have broad-spectrum antibacterial activities against multidrug-resistant pathogens such as Acinetobacter baumannii and *K. pneumoniae* [130]. Tleubayeva et al. (2021) further demonstrated that carbon dioxide extracts of purslane possess antibacterial properties against *E. coli*, *S. aureus*, *B. subtilis*, *and* antifungal activity against *C. albicans* [46,131].

### 4.8. Reichardia tingitana

The methanol extract of *R. tingitana* demonstrated antibacterial activity against *S. aureus*, *Staphylococcus xylosus* (*S.xylosus*), and *E. coli.* Gas chromatography–mass spectrometry analysis identified 17 chemical components, with 6,10,14-trimethylpentadecan-2-one (21.98%) and methyl oleate (27.26%) the primary constituents. The major chemical classes included hydrocarbons (23.82%), fatty acids and their esters (57.46%), steroids (17.26%), and terpenes (1.48%).

In addition to its antimicrobial properties, *R. tingitana* exhibits other biological activities. Its shoots demonstrate larvicidal effectiveness against *Aedes aegypti* (a dengue virus vector) and possess antioxidant, antibacterial, and cytotoxic properties [47]. In a study by Salama et al. (2022), methanolic extracts tested at 10 mg/L against multiple bacterial strains using the agar disk diffusion assay showed pronounced growth inhibition for *Salmonella typhimurium* (*S. typhimurium*) and *B. cereus*, with inhibition zones of 25.71 ± 1.63 mm and 24.42 ± 0.81 mm, respectively [47]. Conversely, *Clostridium tetani* and *S.xylosus* exhibited the highest resistance.

The antibacterial activity of *R. tingitana* against *B. cereus*, *E. coli*, and *S. typhimurium* was comparable to that of standard antibiotics, corroborating findings from earlier studies on essential oils and n-hexane extracts of the plant’s aerial parts. These studies demonstrated potent activity against both Gram-positive and Gram-negative bacteria, with a particular efficacy against Gram-negative species.

These findings underscore the potential of *R. tingitana* as a natural source of bioactive compounds for antimicrobial and therapeutic applications. The broad-spectrum activity of its extracts highlights the need for further investigation into its potential for developing new antimicrobial agents.

### 4.9. Salvadora persica

*S. persica* is a perennial plant [132] traditionally used for oral hygiene due to its significant antibacterial properties [133,134,135]. The effectiveness of *S. persica* is primarily attributed to benzyl isothiocyanate [52,136,137], which inhibits lactic acid production and the growth of *S. mutans*, a key contributor to dental caries [53]. Essential oils from both dried and fresh roots of *S. persica* exhibit strong antibacterial and antibiofilm activities. Fresh root oils contain higher concentrations of benzyl isothiocyanate, comparable to the efficacy of chlorhexidine, a standard oral hygiene agent [52]. Methanolic extracts of *S. persica* have shown potential in endodontic therapy by eradicating *E. faecalis* from root canals, suggesting its utility as an alternative to calcium hydroxide [54]. These findings emphasize the potential of *S. persica* as a natural antimicrobial agent for oral health.

### 4.10. Solanum lycopersicum

*S. lycopersicum* contains bioactive compounds such as carotenoids, flavonoids, and phenolic acids [46,138,139], which exhibit antimicrobial and antioxidant properties [46,84,140]. Tomato lectins (TCLs) have been shown to inhibit bacterial growth, particularly against *Shigella boydii*, *Shigella dysenteriae*, and *S. aureus* [141]. Additionally, studies on tomato processing waste revealed moderate antimicrobial activity against *S. aureus*, correlating with its isochlorogenic acid content [59]. These findings highlight the potential of tomatoes and their by-products as sources of bioactive compounds for antimicrobial applications. Furthermore, the presence of these bioactive compounds in tomatoes suggests their potential use in developing natural antimicrobial agents for various applications in the food and pharmaceutical industries. Overall, tomatoes offer a promising avenue for harnessing their antimicrobial properties through the extraction and utilization of these beneficial compounds.

### 4.11. Trigonella foenum-graecum

*T. foenum-graecum* is rich in bioactive compounds such as saponins, polyunsaturated fatty acids, and trigonelline [142,143,144] contributing to its antimicrobial properties [145,146,147]. Ethanolic extracts of fenugreek have shown significant antibacterial activity against *S. aureus* and *P. aeruginosa* [148]. Additionally, naringenin, a compound found in fenugreek, exhibits antifungal and antimicrobial properties [149]. Fenugreek’s bioactive constituents position it as a promising natural agent for combating infections and managing metabolic disorders [150]. Overall, the diverse bioactive compounds present in fenugreek make it a versatile and effective natural remedy for various health conditions.

### 4.12. Withania somnifera

*W. somnifera* is a medicinal plant with a rich phytochemical profile [66,151], including withanolides, alkaloids, and steroidal saponins, which contribute to its antimicrobial properties [68,69]. The plant has demonstrated activity against methicillin-resistant *S. aureus*, *E. coli*, and *P. aeruginosa.* Additionally, it shows antifungal properties against *C. albicans* and other pathogens [68]. The antimicrobial mechanism disrupts bacterial cell membranes and induces apoptosis-like death in parasites [152,153]. These findings underscore the therapeutic potential of *W. somnifera* in combating infectious diseases. Furthermore, studies have shown that *W. somnifera* may also enhance the immune system’s response to infections, making it a promising natural remedy for various microbial illnesses [154,155]. Continued research into the plant’s antimicrobial properties could lead to the development of new treatments for drug-resistant pathogens.

### 4.13. Ziziphus lotus

*Z. lotus* contains diverse bioactive compounds, including flavonoids, tannins, and saponins [72,156], which contribute to its antimicrobial properties [157,158]. Acetonic extracts of *Z. lotus* leaves have demonstrated efficacy against methicillin-resistant *S. aureus* and other pathogens, with MIC values ranging from 250 to 1000 µg/mL [73]. Furthermore, methanolic and ethanolic extracts show broad-spectrum activity against *E. coli*, *P. aeruginosa*, and *L. monocytogenes.* These findings highlight the potential of *Z. lotus* as a source of natural antimicrobial agents for therapeutic and food safety applications [156,159]. Also, *Z. lotus* extracts have been found to exhibit antioxidant properties [160], which can help in reducing oxidative stress and inflammation in the body [161,162]. This dual functionality makes *Z. lotus* a promising candidate for further research and development in the field of natural medicine and food preservation (Table 1). 

### 4.14. Effect Size of Medicinal Plants for Different Antibacterial Activities

**Figure 1:**
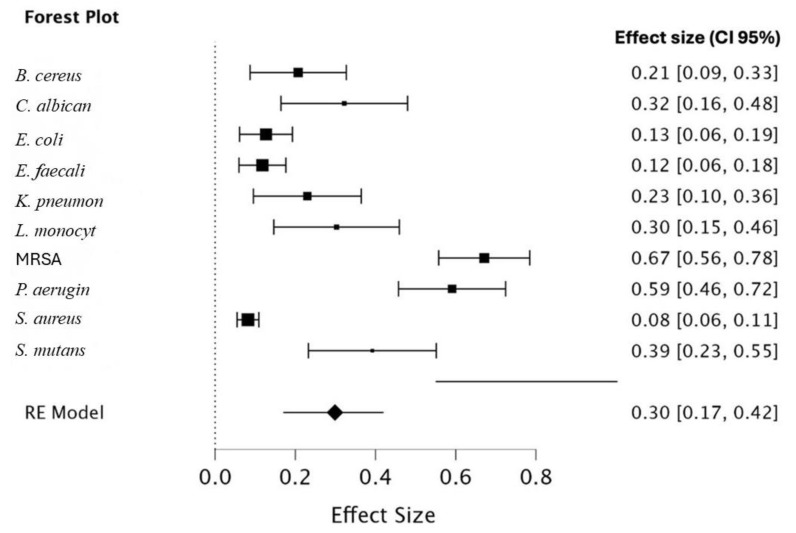
Forest plot presenting the antimicrobial activities against various selected bacterial pathogens from selected medicinal plant species. Figure and analysis produced by JASP statistical software 2024 version 0.19 for Mac OS. Figure supported with effect size, confidence interval both upper and lower limits. *E. coli*, *Escherichia coli*; *S. aureus*, *Staphylococcus aureus*; *C. albicans*, *Candida albicans*; MRSA, methicillin-resistant *S. aureus*; *B. cereus*, *Bacillus cereus*; *K. pneumoniae*, *Klebsiella pneumoniae*; *S. mutans*, *Streptococcus mutans*; *E. faecalis*, *Enterococcus faecalis*; *L. monocytogenes*, *Listeria monocytogenes*; *P. aeruginosa*, *Pseudomonas aeruginosa.*

The results of this study indicate varying levels of antibacterial activity of the selected medicinal plants against the tested pathogens, as reflected by the effect size (ES) (η^2^), confidence intervals, and corresponding *p*-values. Notably, significant effects were observed against *E. coli* (η^2^ = 0.127, *p* = 0.005), *E. faecalis* (η^2^ = 0.118, *p* < 0.001), *MRSA* (η^2^ = 0.671, *p* < 0.001), *S. aureus* (η^2^ = 0.082, *p* = 0.009), and *S. mutans* (η^2^ = 0.392, *p* < 0.001). Among these, MRSA demonstrated the highest ES (η^2^ = 0.671), suggesting a strong antibacterial response, while *S. mutans* also exhibited a considerable effect (η^2^ = 0.392). In contrast, non-significant results were observed for *B. cereus*, *C. albicans*, *K. pneumoniae*, *L. monocytogenes*, and *P. aeruginosa*, despite some showing moderate effect sizes, such as *L. monocytogenes* (η^2^ = 0.303) and *P. aeruginosa* (η^2^ = 0.591). The high ES observed for *P. aeruginosa* but its lack of statistical significance (*p* = 0.626) suggests variability in the data. Overall, the findings highlight the potential of selected medicinal plants to exhibit strong antibacterial activity, particularly against MRSA and *S. mutans*, which could be promising targets for further research in antimicrobial applications (Table 2, Figure 1).

The high ES observed for the antibacterial activity of selected medicinal plants, particularly against MRSA (η^2^ = 0.671, *p* < 0.001) and *S. mutans* (η^2^ = 0.392, *p* < 0.001), highlights their potential as effective alternatives to conventional antibiotics. These findings are comparable to studies on other herbal remedies, such as the work by Christensen et al. (2008) [166], which demonstrated that rose hip exhibited an ES superior to or comparable with that of NSAIDs, resulting in reduced reliance on medications associated with significant side effects. Similarly, our results suggest that these plants may reduce the need for antibiotics, especially for resistant pathogens like MRSA or in applications such as oral healthcare for *S. mutans*. This is particularly important considering the widespread adverse effects and economic burden associated with conventional antibiotics. Additionally, leveraging these plants could contribute to mitigating the global issue of antimicrobial resistance (AMR). While some pathogens, such as *P. aeruginosa*, demonstrated high ES but non-significance, this variability warrants further investigation to validate these findings. Future research should focus on clinical trials to evaluate whether the observed ES translates into reduced consumption of rescue or prescription medications, ultimately benefiting both public health and healthcare costs.

## 5. Overview of Gastrointestinal Disease with Medicinal Plants in the UAE

### 5.1. Allium cepa

The utilization of natural compounds for managing gastrointestinal disorders, particularly gastritis and ulcerative conditions, is increasingly supported by scientific evidence. Notably, the gastroprotective potential of *A. cepa* red onion peel extract has been demonstrated in various studies, showing significant efficacy in reducing the ulcer index in ethanol-induced gastric injuries in rats. These effects are attributed to the modulation of critical inflammatory pathways such as Nrf2/HO-1 and HMGB-1/NF-κB [167]. Furthermore, the broad therapeutic applications of *A. cepa*, encompassing anti-inflammatory, antioxidant, and antimicrobial properties, substantiate its potential against a range of disorders, including gastrointestinal ailments [28,29]. The presence of organosulfur compounds in onions has been identified as a critical factor in these health benefits, showing promise in modulating gut microbiota and mitigating inflammatory responses in the gastrointestinal tract [168].

Additionally, studies have highlighted the specific actions of these compounds in the prevention of natural or chemical toxicities, further supporting their role in gastroprotection [169]. The evidence extends to the effectiveness of onion bulb extract in reducing the severity of colitis in mice, thus reinforcing the potential of onion-derived compounds in treating inflammatory bowel diseases [170]. Furthermore, the antidiarrheal properties of *A. cepa* have been confirmed in preclinical studies, demonstrating its ability to inhibit gastrointestinal motility similar to standard drugs like loperamide [171]. These findings collectively highlight the therapeutic versatility of *A. cepa*, suggesting its substantial potential in managing and treating a wide range of gastrointestinal disorders, thus warranting further investigation and validation in clinical settings.

### 5.2. Allium sativum

Garlic oil, rich in diallyl and allyl methyl sulfides, has shown significant effectiveness against *H. pylori* [81]. Allicin, a key compound, can react with itself to form ajoene, which further enhances garlic’s antimicrobial action. These compounds disrupt bacterial cell functions, including membrane integrity, enzyme activity, and metabolic processes, making garlic a promising natural agent for combating bacterial infections, including multidrug-resistant strains [81].

Garlic oil has demonstrated antibacterial effects against pathogens like *S. aureus*, *E. coli*, and *Bacillus subtilis*, and clinical trials have shown its ability to suppress *H. pylori* in the stomach, offering therapeutic potential for infection treatment [172]. Garlic extracts, particularly allicin, also disrupt bacterial cell membranes, making them effective against antibiotic-resistant strains like *S. aureus* [173]. Furthermore, the combination of garlic and ginger has been shown to have a synergistic antibacterial effect, with the most significant inhibition observed against *Mycobacterium tuberculosis* and *E. coli* [174]. These findings underscore garlic’s potential as a natural, effective antimicrobial agent for treating a variety of GI infections.

### 5.3. Cymbopogon schoenanthus

Recent studies have highlighted the multifaceted applications of *Cymbopogon* species, emphasizing their traditional usage and potent bioactive properties. Mokhtar et al. (2023) demonstrated that the methanolic extract of *C. schoenanthus* exhibits significant antibacterial activity against foodborne pathogens, further supported by detailed phytochemical analysis that revealed the presence of various bioactive compounds such as tannins and terpenoids [37]. Complementing this, Zhao et al. (2024) provided a comprehensive review of the ethnobotanical uses and pharmacological activities of *Cymbopogon* plants, documenting over 150 isolated compounds with known therapeutic effects such as anti-inflammatory and antidiabetic activities [175]. Similarly, Gomes et al. (2017) investigated the anti-inflammatory potential of phenol-rich extracts from *Cymbopogon citratus* and *C. schoenanthus*, finding significant inhibition of proinflammatory markers in cellular models [176]. Additionally, a review by Bossou et al. (2020) details the diverse biological properties of essential oils from *Cymbopogon* species, underscoring their utility in traditional and modern applications across various industries [177]. Lastly, Tibenda et al. (2022) summarize the extensive pharmacological activities associated with the *Cymbopogon* genus, advocating for further research to validate its use in phytotherapy and pharmaceuticals [178]. These studies collectively enhance our understanding of *Cymbopogon*’s potential in medical and industrial applications, presenting a strong case for its continued investigation and integration into health therapies.

### 5.4. Lawsonia inermis

Recent investigations have extensively explored the therapeutic potential of *L.inermis*. Sultana and Khosru, (2011) evaluated the analgesic and antidiarrheal activities of its leaf extracts, noting moderate analgesic effects but significant antidiarrheal properties at a dose of 500 mg/kg [179]. Complementing this, Goswami et al. (2011) and Mohammed et al. (2022) demonstrated its anti-ulcer efficacy in rat models, showing that both traditional and nano-formulated extracts effectively reduce ulcer indices [180,181]. Trigui et al. (2013) further highlighted the antibacterial efficacy of its leaves against plant pathogens, indicating potential for agricultural applications [182]. Lastly, an extensive review by Batiha et al. (2024) provided a comprehensive overview of its bioactive components and diverse pharmacological activities, reinforcing the plant’s utility in treating a variety of ailments and its potential in drug development [183]. These studies collectively affirm the substantial pharmacological and therapeutic benefits of *L. inermis*, supporting its traditional and potential modern medical uses.

### 5.5. Matricaria aurea

Recent studies have emphasized the efficacy and safety of herbal medicines in treating gastrointestinal disorders, focusing on a medicinal product containing myrrh, chamomile, and coffee charcoal. Albrecht et al. (2014) conducted a non-interventional study illustrating the significant improvement in symptoms of acute diarrhea among patients with various gastrointestinal disorders, highlighting its effectiveness across treatment modalities, including monotherapy and as part of combination therapy [184]. Complementing this, Gupta (2010) reviewed the extensive historical and medicinal background of chamomile, detailing its application in treating a wide range of ailments, including gastrointestinal disorders, which underscores the herb’s broad therapeutic potential [185]. Al-Hashem (2010) demonstrated the gastroprotective effects of chamomile against ethanol-induced ulcers in rats, presenting a strong case for its use in preventive strategies for gastric ulcers [186]. Additionally, Bezerra et al. (2009) explored the mechanisms behind the gastroprotective effects of α-bisabolol, a component of chamomile, showing significant ulcer reduction facilitated by the activation of K+ATP channels, which highlights the intricate biochemical pathways involved in its therapeutic action [187]. These findings collectively support the integration of these herbal components into treatment regimens for gastrointestinal health, providing a natural, effective, and well-tolerated option for patients suffering from various gastrointestinal ailments.

### 5.6. Nigella sativa

Recent studies have significantly contributed to the understanding of the therapeutic potential of *N. sativa* in treating gastrointestinal diseases. Jarmakiewicz-Czaja et al. (2023) highlighted the diverse medicinal benefits of *N. sativa*, particularly its bioactive compound thymoquinone, which exhibits notable hypoglycemic, hypolipemic, and hepatoprotective effects [188]. Alizadeh-naini et al. (2020) demonstrated its efficacy in enhancing *H. pylori* eradication rates when combined with conventional therapy, significantly improving patients’ quality of life [189]. Yousefnejad et al. (2023) further explored *N. sativa*’s role in increasing serum ghrelin levels and appetite among *H. pylori*-infected patients, underscoring its potential as an adjunctive therapy [190]. Shakeri et al. (2016) reviewed the gastrointestinal effects of *N. sativa* and thymoquinone, noting their ability to control acid secretion and protect against gastric ulcers [191]. Additionally, Tayman et al. (2012) confirmed the beneficial effects of *N. sativa* oil on intestinal damage in necrotizing enterocolitis, highlighting its protective properties in severe intestinal conditions [192]. The collective findings from these studies enhance our understanding of *N. sativa*’s gastroprotective mechanisms and reinforce its integration into clinical practices to mitigate various gastrointestinal disorders effectively.

### 5.7. Phoenix dactylifera

Recent systematic reviews and clinical trials have highlighted the multifaceted therapeutic benefits of *P. dactylifera*, particularly in managing gastrointestinal disorders. Bagherzadeh Karimi et al. (2020) conducted a systematic review of clinical trials exploring the effects of various botanical parts of date palm across multiple medical specialties, including gastroenterology, emphasizing the need for further high-quality randomized controlled trials to solidify these findings [193]. Metwally et al. (2022) demonstrated in a randomized clinical trial that regular intake of date fruits significantly alleviated gastrointestinal symptoms in autistic children, suggesting dates’ potential to improve gastrointestinal health [107]. Eid et al. (2015) found that consumption of dates did not significantly change the growth of colonic microbiota, but did reduce colonic genotoxicity and improve bowel movements, indicating a potential role in colorectal cancer prevention [194]. Camilleri et al. (2023) reviewed the anti-constipation effects of dates, identifying their high fiber and bioactive chemical content as key factors in promoting laxative properties [195]. Lastly, Al-Qarawi et al. (2005) explored the gastroprotective effects of dates against ethanol-induced gastric ulcers in rats, noting their ability to mitigate ulcer severity through antioxidant actions, which may also underpin their therapeutic benefits in humans [196]. These studies collectively underscore the potential of date palm as a natural and effective remedy for a range of gastrointestinal ailments, supporting its inclusion in dietary recommendations and therapeutic interventions.

### 5.8. Portulaca oleracea

Recent studies have underscored the significant therapeutic potential of *Portulaca oleracea*, particularly in the context of digestive inflammatory conditions and constipation. Shao et al. (2024) reviewed the use of *P. oleracea* in traditional Chinese medicine for the treatment of digestive system cancers and inflammation-related conditions, highlighting its anti-inflammatory and cancer-modulating effects, mainly through compounds such as kaempferol and quercetin [197]. Bang et al. (2022) demonstrated the efficacy of *P. oleracea* in managing functional constipation in a randomized, double-blind, placebo-controlled trial, noting significant improvements in bowel movement frequency and quality of life among treated patients [198]. Zhang et al. (2022) explored *P. oleracea*’s mechanism in alleviating intestinal inflammation by regulating endoplasmic reticulum stress and autophagy pathways, providing insight into its protective effects in inflammatory bowel disease (IBD) models [199]. Iranshahy et al. (2017) provided a comprehensive overview of the phytochemistry and pharmacological properties of *P. oleracea*, discussing its traditional and pharmacological applications in treating a wide range of diseases, including gastrointestinal disorders [200]. Lastly, Kim et al. (2018) highlighted the anti-inflammatory properties of *P. oleracea* extracts in vitro and in vivo, showing their efficacy in reducing cytokine production and ameliorating symptoms in a mouse model of ulcerative colitis, suggesting *P. oleracea* as a promising natural treatment for IBD [201]. Collectively, these studies reinforce the potential of *P. oleracea* as an effective natural remedy for a range of gastrointestinal ailments, meriting further clinical exploration.

### 5.9. Salvadora persica

Recent studies have highlighted the significant therapeutic benefits of *S. persica* in managing gastric ulcers and other digestive disorders. Lebda et al. (2018) investigated the protective effects of *S. persica* extract against ethanol-induced gastric ulcers in rats, demonstrating its ability to alleviate gastric hemorrhagic necrosis, modulate oxidative stress, and downregulate proinflammatory cytokines, suggesting a potential mechanism through antioxidant defense and anti-inflammatory action [202]. Filimban et al. (2015) assessed the aqueous extracts of miswak in treating aspirin-induced gastric ulcers in rats, noting significant ulcer healing and a decrease in histopathological damage, which aligns with its traditional use for stomach health [203]. Finally, Ahmad et al. (2012) discussed the extensive ethnobotanical applications and pharmacological potential of miswak, underscoring its role in traditional medicine across different cultures and its diverse pharmacological actions, supporting its use as a natural remedy for a variety of ailments [204]. These studies underscore *S. persica*’s potential as an effective natural treatment for gastric ulcers and other gastrointestinal issues, highlighting the need for further clinical trials to explore and validate its therapeutic properties fully.

### 5.10. Solanum lycopersicum

Recent research highlights the significant pharmacological potential and dietary benefits of *S. lycopersicum*, underscoring its value both in traditional medicine and contemporary nutritional applications. Rawat et al. (2024) provided a detailed review of the tomato plant, discussing its botanical characteristics, chemical constituents, and a wide range of traditional and modern pharmacological uses. The review emphasized tomatoes’ therapeutic properties, particularly anti-inflammatory and antioxidant activities, which contribute to their role in preventing various chronic diseases [205]. Complementing this, Jiang and Shao (2024) analyzed data from the National Health and Nutrition Examination Survey to explore the relationship between vegetable intake and constipation incidence, identifying non-starchy vegetables, especially tomatoes, as being significantly associated with a reduction in constipation risk among the general U.S. population [206]. Their findings highlight the specific benefits of tomatoes in promoting gastrointestinal health, distinguishing them from other starchy vegetables that did not show similar benefits. Together, these studies provide robust evidence supporting the inclusion of tomatoes in the diet for their health-promoting properties, particularly in enhancing digestive health and preventing constipation, while offering broader therapeutic applications.

### 5.11. Trigonella foenum-graecum

*T. foenum-graecum* has demonstrated significant pharmacological properties and therapeutic benefits, particularly in gastrointestinal health. Research conducted by Zarghi et al. (2021) assessed fenugreek’s efficacy in reducing gastrointestinal bleeding in mechanically ventilated patients [207]. However, the findings showed that while there was a reduction, it was not statistically significant. Complementarily, Selmi et al. (2022) and Singaravelu et al. (2018) investigated fenugreek’s gastroprotective effects against experimentally induced gastric ulcers in rats, revealing significant ulcer healing and anti-ulcer potential through modulation of oxidative stress markers and enhancement of antioxidant enzyme activities, illustrating its protective properties [208,209]. Further emphasizing its gastrointestinal benefits, studies like those by Kheirandish et al. (2011) have shown that pre-treatment with fenugreek can protect against intestinal ischemia–reperfusion injury in rats, underscoring its potential as a powerful herbal remedy in gastroenterology [210].

### 5.12. Withania somnifera

*Withania somnifera* (*W. somnifera*) demonstrates extensive therapeutic benefits across various clinical and experimental settings. Research by Punukollu et al. (2024) demonstrated that a proprietary blend of ashwagandha root and okra fruit extracts effectively relieves constipation and enhances bowel function, as evidenced by significant improvements in PAC-SYM, PAC-QOL, and GSRS scores [211]. Complementarily Pawar et al. (2011) highlighted its much-restorative and anti-inflammatory efficacy in inflammatory bowel disease, reinforced by improvements in histopathological parameters and antioxidant enzyme activities [212]. A subsequent study by Singh et al. (2024) supported these findings, where supplementation not only relieved functional constipation but also improved gastrointestinal function and reduced inflammatory markers in a larger cohort [213]. These pharmacological activities are attributed to its rich content of secondary metabolites, such as withanolide alkaloids, which Saleem et al. (2020) noted for their anti-inflammatory effects in the GI tract. This broad spectrum of benefits, coupled with its adaptogenic effects, positions *W. somnifera* as a versatile botanical with significant clinical relevance for managing digestive and inflammatory conditions and enhancing overall health and wellness [66].

### 5.13. Ziziphus lotus

**Figure 2:**
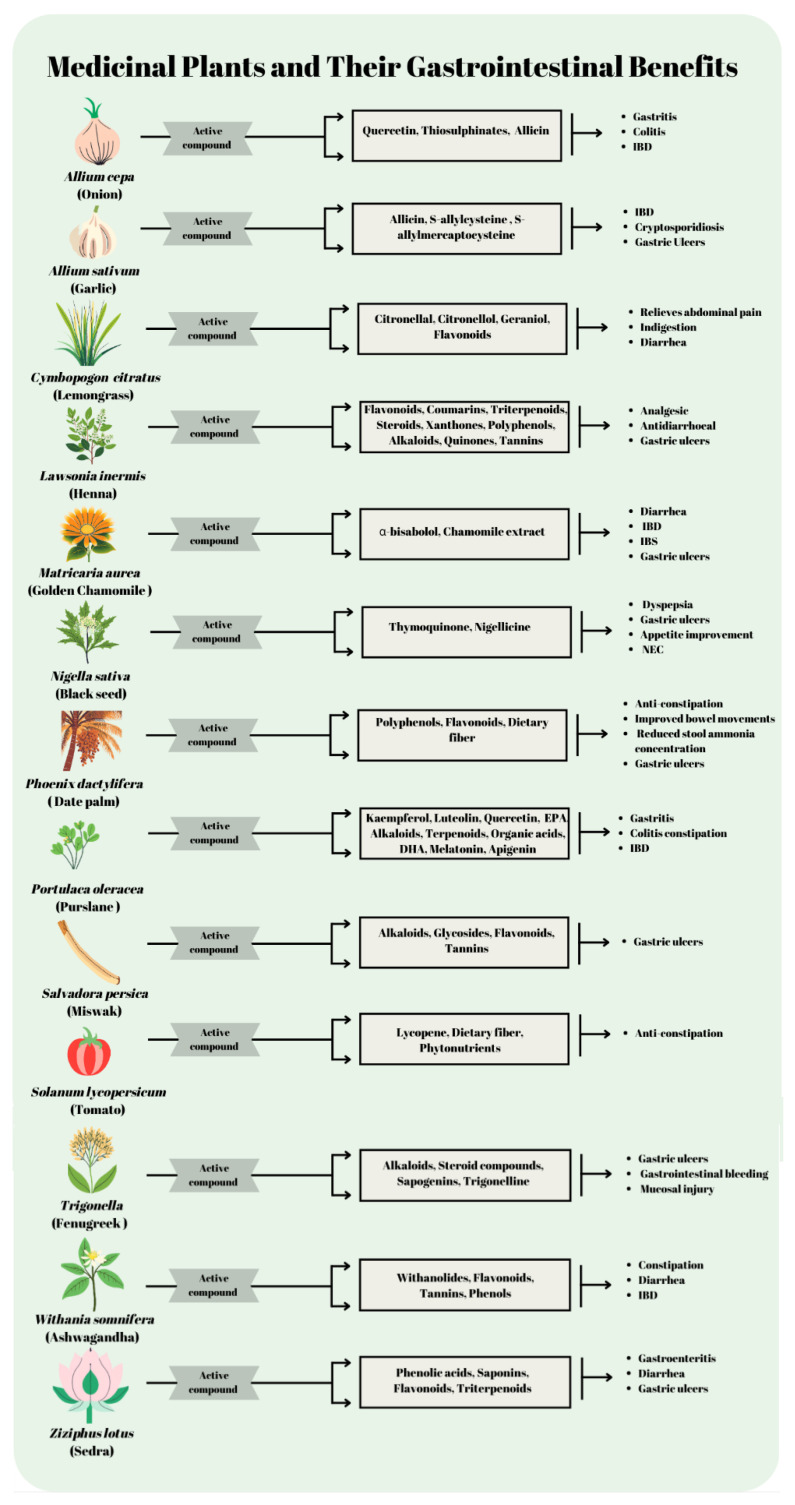
Medicinal plant and their gastrointestinal benefits, IBD, inflammatory bowel disease; IBS, irritable bowel syndrome; NEC, necrotizing enterocolitis.

Due to its significant therapeutic properties, *Z. lotus* is prominent in traditional and contemporary medical applications. Bekkar et al. (2023) demonstrated the effective anti-gastroenteritis properties of *Z. lotus* against *S. enterica* with a notable reduction in liver enzyme levels, indicating potential use in developing antibacterial treatments [214]. Additionally, Bakhtiar et al. (2024) and Wahida et al. (2007) revealed *Z. lotus*’s gastroprotective capabilities and its efficacy against *H. pylori*, reinforcing its use in treating ulcers and other gastrointestinal disorders [143,215]. Agrawal et al. (2023) and Mesmar et al. (2022) further explored the phytochemical diversity of the *Ziziphus* genus, highlighting its broad pharmacological spectrum and advocating its inclusion in dietary regimes to combat various ailments [216,217]. Hani et al. (2020) supported these findings by demonstrating *Z. lotus*’s anti-inflammatory and antidiarrheal activities in vivo, providing empirical support for its traditional use in herbal medicine [218]. Lastly, Sakna et al. 2023 provided a detailed review of the triterpenes from the *Ziziphus* genus, underscoring their potential as bioactive components in modern therapeutic application [219]. Collectively, these studies emphasize the potential of *Ziziphus* species in pharmaceutical developments for gastrointestinal and infectious diseases, backed by their rich phytochemical profiles and pharmacological benefits (Figure 2).

### 5.14. Effect Size of Medicinal Plants for Different GIT Disorder Treatments

The results indicate the effect size (η^2^) of treatments with 13 selected plant species for gastrointestinal tract (GIT) disorders. Among the measured outcomes, constipation showed the largest effect size (η^2^ = 0.449, SE = 0.123), though not statistically significant (*p* = 0.135), followed by abdominal pain (η^2^ = 0.339, SE = 0.092, *p* = 0.348) and gastric ulcers (η^2^ = 0.316, SE = 0.085, *p* = 0.412). Similarly, GIT bleeding (η^2^ = 0.315), IBS (η^2^ = 0.315), and GIT improvement (η^2^ = 0.313) exhibited moderate effect sizes with overlapping confidence intervals, indicating consistency in their outcomes, though none achieved statistical significance.

**Figure 3:**
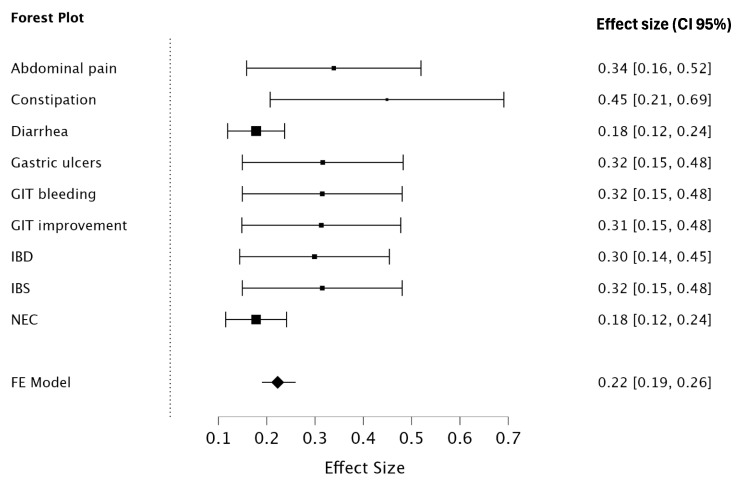
Forest plot presenting the GIT disorder treatments with 13 selected plant species. Figure and analysis produced by JASP statistical software 2024 version 0.19 for Mac OS. Figure supported with effect size, confidence interval, both upper and lower limits. IBD, inflammatory bowel disease; IBS, irritable bowel syndrome; NEC, necrotizing enterocolitis.

The forest plot further highlights constipation as the condition with the highest effect size, with a confidence interval ranging between 0.21 and 0.69, followed by abdominal pain and gastric ulcers. In contrast, conditions like diarrhea (η^2^ = 0.178) and NEC (η^2^ = 0.178) displayed smaller effect sizes, with tighter confidence intervals. Overall, while the treatments showed promising moderate effect sizes across several GIT disorders, the non-significant *p*-values suggest that further investigations with larger samples are warranted to confirm these trends and establish statistical significance (Table 3, Figure 3).

The findings from this study indicate that the selected plant species exhibit varying levels of efficacy in treating gastrointestinal tract (GIT) disorders, as measured by effect size (η^2^). Among the evaluated conditions, constipation demonstrated the highest effect size (η^2^ = 0.449, SE = 0.123), followed by abdominal pain (η^2^ = 0.339, SE = 0.092) and gastric ulcers (η^2^ = 0.316, SE = 0.085). Although none of these results achieved statistical significance, the moderate effect sizes suggest potential therapeutic benefits. For instance, constipation, with an effect size ranging between 0.21 and 0.69, could be a promising target for further research on herbal treatments. Similarly, GIT bleeding (η^2^ = 0.315), IBS (η^2^ = 0.315), and GIT improvement (η^2^ = 0.313) showed consistent moderate effect sizes, highlighting their potential for addressing these conditions.

Drawing parallels with established herbal remedies, such as rose hip for osteoarthritis pain management, the high effect sizes observed in this study raise the possibility of reducing the reliance on conventional prescription medications. For example, the use of herbal treatments with comparable or superior effect sizes to over-the-counter or prescription medications could mitigate the side effects and high costs associated with standard drugs. This could have significant implications for public health and healthcare costs, particularly if further studies demonstrate statistical significance and clinical efficacy for these herbal treatments.

The observed effect sizes in this study suggest potential benefits that, while impactful, should be carefully distinguished from those expected of conventional pharmaceuticals. These medicinal plants, commonly incorporated into the diet, may offer incremental health benefits and represent a less intensive approach to disease management compared to prescription medications. This distinction is crucial, as it reflects the gradual and supportive role of dietary interventions in health management, rather than the acute, sometimes drastic, effects seen with pharmaceuticals. Furthermore, while the therapeutic potential of these plants is promising, it is essential to consider their complex nature and the possibility of side effects or allergic reactions, as evidenced by reports of such occurrences in this review.

While the non-significant *p*-values underscore the need for larger samples and more robust study designs [166], the results suggest that certain GIT disorders, particularly constipation and abdominal pain, may benefit from herbal interventions. These findings align with the broader context of integrating herbal remedies into mainstream healthcare, where they could serve as adjunct or alternative treatments, potentially reducing the burden of rescue or prescription medications. Future research should focus on confirming these trends through well-designed clinical trials, exploring both the therapeutic and economic implications of using herbal treatments for GIT disorders.

## 6. Conclusions

This thorough analysis of medicinal plants in the UAE has highlighted their essential role and significant potential in tackling various health issues, including diabetes, cardiovascular diseases, and antibiotic-resistant infections. The efficient utilization of these indigenous plants enhances therapeutic results and aligns with sustainable healthcare practices by leveraging local biodiversity. Moreover, the economic and societal implications of integrating these herbal remedies into mainstream healthcare are profound, as they may reduce reliance on medications with significant side effects, lower healthcare costs, and mitigate the challenges of antimicrobial resistance.

In evaluating the medicinal plants, we considered their popularity and the extent of available research. Globally widespread plants like onion (*Allium cepa*) and garlic (*Allium sativum*) are extensively studied due to their universal culinary and medicinal applications. Conversely, local plants such as the date palm (*Phoenix dactylifera*), integral to Middle Eastern cultures, might not have the same volume of global research, but are deeply embedded in regional medicinal practices.

Notwithstanding the encouraging outcomes, substantial challenges must be addressed to optimize the therapeutic potential of these plants. Standardization of extracts, validation of traditional claims through rigorous scientific studies, and sustainable harvesting practices are essential to ensure the safety, efficacy, and continuity of plant-based therapies. Future research should focus on clinical trials to confirm these findings, evaluate their practical applications, and explore their broader therapeutic and economic impact.

In light of the findings, it is clear that herbal remedies not only offer an alternative therapeutic option but also align with global health economic strategies aimed at reducing reliance on conventional pharmaceuticals. The integration of these traditional treatments could substantially lower healthcare costs and minimize the side effects associated with many modern medications. As such, the broader application of herbal remedies promises not just health benefits but also economic advantages by fostering more sustainable healthcare practices worldwide. This shift could significantly impact public health policy, encouraging the inclusion of effective, economically viable, and less invasive treatment options in standard healthcare protocols.

## 7. Future Perspectives

Future integration of advanced pharmacological assessments, molecular biology methodologies, and nanotechnology could substantially enhance the efficacy of medicinal plants. These technologies may assist in identifying and isolating the most efficacious compounds with minimal adverse effects. Additionally, cultivating partnerships between local knowledge holders, researchers, and pharmaceutical industries can foster a translational approach that ensures the practical application of research findings. Sustainable practices must be a cornerstone of future explorations to protect these valuable botanical resources, supporting local ecosystems and global health.

## Figures and Tables

**Table 1:** Antibacterial properties of medicinal plants in the UAE.

Medicinal Plant (Used Part)	Activity Against Pathogens										References
	***S. aureus***	***E. coli***	***C. albicans***	**MRSA**	***B. cereus***	***K. pneumoniae***	***S. mutans***	***E. faecalis***	***L. monocytogenes***	***P. aeruginosa***	
***A. cepa* (onion)** **(onion peel)**	+	−	−	−	−	−	−	−	−	−	[15]
***A. sativum* (garlic)** **(Pulp)**	+	+	+	+	−	−	−	−	+	+	[81]
***Capparis spinosa*** **(fruit flower, stem, shoots roots)**	+	+	−	−	−	−	−	−	−	−	[163]
***C. schoenanthus* (lemongrass)** **(methanolic extract)**	+	+	−	+	+	−	−	−	−	−	[37,98]
***L. inermis* (henna)** **(leaves, juice of leaves, oil extract)**	+	+	+	−	−	+	−	−	−	−	[105]
***M. aurea* (golden chamomile)** **(EO from *M. aurea*)**	+	−	−	−	−	−	−	−	−	+	[109]
***P. dactylifera* (date palm)** **(palm fruit)**	+	+	+	−	+	−	−	−	−	−	[121]
***P. oleracea* (purslane)** **(roots)**	+	+	+	−	−	+	−	+	−	+	[127]
***R. tingitana* (poppy-leaved)** **(shoot extract)**	+	+	−	−	+	−	−	−	−	−	[47]
***S. persica* miswak)** **(roots, twigs, or stems)**	+	−	−	−	−	−	+	+	−	−	[52,164]
***S. lycopersicum* (tomato)** **(fruit)**	+	−	−	−	−	−	−	−	−	−	[141]
***Trigonella* (fenugreek)** **(seeds)**	+	+	−	−	−	−	−	−	−	+	[35,61,62]
***W. somnifera* (ashwagandha)** **(fruits, leaves, roots)**	+	+	+	−	−	−	−	−	−	+	[67,69]
***Z. lotus* (sedra)** **(leaves, branches, fruit and root and stem barks)**	+	+	−	+	−	−	−	−	+	+	[70,72,165]

*E. coli*, *Escherichia coli*; *S. aureus*, *Staphylococcus aureus*; *C. albicans*, *Candida albicans*; MRSA, methicillin-resistant *S. aureus*; *B. cereus*, *Bacillus cereus*; *K. pneumoniae*, *Klebsiella pneumoniae*; *S. mutans*, *Streptococcus mutans*; *E. faecalis*, *Enterococcus faecalis*; *L. monocytogenes*, *Listeria monocytogenes*; *P. aeruginosa*, *Pseudomonas aeruginosa;* EO, essential oil. "+" denotes observed antibacterial activity of plant extracts against pathogens, while "−" indicates no observed activity.

**Table 2:** Effect size in terms of eta squared (η^2^) for different antibacterial activities of selected 13 plants and study pathogens.

Pathogen	Point Estimate	95% Confidence Interval		*p*-Value
		Lower	Upper	
*B. cereus*	0.207	0.000	0.240	0.872 ns
*C. albicans*	0.322	0.056	0.372	0.553 ns
*E. coli*	0.127	0.000	0.132	0.005 **
*E. faecalis*	0.118	0.000	0.117	<0.001 ***
*K. pneumoniae*	0.230	0.000	0.269	0.123 ns
*L. monocytogenes*	0.303	0.039	0.352	0.069 ns
MRSA	0.671	0.482	0.709	<0.001 ***
*P. aeruginosa*	0.591	0.369	0.636	0.626 ns
*S. aureus*	0.082	0.000	0.054	0.009 **
*S. mutans*	0.392	0.126	0.445	<0.001 **

Significant differences in levels: *p* < 0.001 ***, *p* < 0.01 **. ns, not significant. *E. coli*, *Escherichia coli*, *S. aureus*, *Staphylococcus aureus*; *C. albicans*, *Candida albicans*; MRSA, methicillin-resistant *S. aureus*; *B. cereus*, *Bacillus cereus*; *K. pneumoniae*, *Klebsiella pneumoniae*; *S. mutans*, *Streptococcus mutans*; *E. faecalis*, *Enterococcus faecalis*; *L. monocytogenes*, *Listeria monocytogenes*; *P. aeruginosa*, *Pseudomonas aeruginosa*.

**Table 3:** Effect size in terms of eta squared (η^2^) for different GIT disorder treatments with 13 selected plant species.

Disease	ES (Eta Squared)	SE	95% Confidence Interval		(*p*-Value)
			Lower	Upper	
Abdominal pain	0.339	0.092	0.0	0.361	0.348 ns
Constipation	0.449	0.123	0.0	0.484	0.135 ns
Diarrhea	0.178	0.030	0.0	0.118	0.858 ns
Gastric ulcers	0.316	0.085	0.0	0.333	0.412 ns
GIT bleeding	0.315	0.084	0.0	0.331	0.416 ns
GIT improvement	0.313	0.084	0.0	0.329	0.422 ns
IBD	0.299	0.079	0.0	0.310	0.465 ns
IBS	0.315	0.084	0.0	0.331	0.416 ns
NEC	0.178	0.032	0.0	0.126	0.845 ns

ns, not significant.
